# Bearded capuchin monkey as a model for Alzheimer’s disease research

**DOI:** 10.21203/rs.3.rs-3495799/v1

**Published:** 2023-12-06

**Authors:** Roberta Diehl Rodriguez, Maria Clotilde Tavares, Sonia Maria Brucki, Leonel Tadao Takada, Maria Concepción Otaduy, Maria da Graça Morais Martin, Cláudia Suemoto, Lea Grinberg, Cláudia Leite, Carlos Tomaz, Ricardo Nitrini

**Affiliations:** Faculdade de Medicina da Universidade de São Paulo; University of Brasília; University of São Paulo; University of São Paulo; Faculdade de Medicina da Universidade de São Paulo; Faculdade de Medicina da Universidade de São Paulo; University of São Paulo; University of São Paulo; Faculdade de Medicina da Universidade de São Paulo; Euro-American University Center – UNIEURO; University of São Paulo

## Abstract

The absence of a natural animal model is one of the main challenges in Alzheimer’s disease research. Despite the challenges of using non-human primates in studies, they can bridge mouse models and humans, as non-human primates are phylogenetically close to humans and can spontaneously develop AD-type pathology. The capuchin monkey, a New World primate, has recently attracted attention due to its skill in creating and using instruments. We analyzed three capuchin brains using structural 7T MRI and neuropathological evaluation. Alzheimer-type pathology was found in one case. Widespread β-amyloid pathology mainly in the form of focal deposits with variable morphology and high density of mature plaques. Noteworthy, plaque-associated dystrophic neurites, associated with disrupted of axonal transport and early cytoskeletal alteration, were frequently found. Unlike other species of New World monkeys, cerebral arterial angiopathy was not the predominant form of β-amyloid pathology. Additionally, abnormal aggregates of hyperphosphorylated tau, resembling neurofibrillary pathology, were observed in the temporal and frontal cortex. Besides, astrocyte hypertrophy surrounding plaques was found, suggesting a neuroinflammatory response. Aged capuchin monkeys can spontaneously develop Alzheimer-type pathology, indicating that they may be an advantageous animal model for research in Alzheimer’s disease.

## One-Sentence Summary

Capuchin monkeys can spontaneously develop Alzheimer-type pathology.

## INTRODUCTION

Alzheimer’s disease (AD) is the main cause of dementia and represents one of the biggest challenges for public health^1^. One significant AD research obstacle is the absence of a natural animal model. A reliable animal model of disease is a mainstay of more effective translational research^2^.

Humans and many mammals show a decline in cognition and behavior associated with aging, but while the cognitive decline in humans is mainly attributed to AD, aging has been assumed to cause the decline in other mammals^3^.

The presence of amyloid in the brains of old mammals has been reported; however, they failed to show NFT pathology^4, 5^. Abnormal hyperphosphorylated tau has been reported in several aged mammal neurons, but most are without classic NFT ^6,7^. A consensus prevailed for several years that although amyloid plaques were present in many aged mammals, NFT was absent, proving that AD was an exclusively human disease^8,9^.

The discovery of the amyloid precursor protein (APP) mutation made it possible to produce a transgenic mouse model of AD^2^. However, it was necessary to include other mutated genes to obtain plaque pathology but, even so, without tau pathology or neurodegeneration^2^. Rosen et al. described NFT pathology and plaque-associated dystrophic neurites in a 41-year-old female chimpanzee^10^. Further, Edler et al. analyzed the brains of 20 chimpanzees aged 37 to 62, observing Aβ in plaques and blood vessels, and tau aggregates in the form of neurofibrillary pathology^10^. In contrast with the previous concept, these results suggest that “AD-like pathology is not limited to the human brain” ^10^.

The chimpanzee could be regarded as the first natural animal model of AD. However, chimpanzees are at high risk of extinction, and housing costs considerably limit their use in research^2^. More recently, hyperphosphorylated tau aggregates were shown in mouse lemurs, and NFTs have been demonstrated in rhesus macaques and vervet monkeys^7,11,12^.

The capuchin monkeys (*Sapajus sp*) are one of South America’s most common nonhuman primates. They inhabit Brazilian savanna-like environments and mangroves in the Northeastern and Central regions^13,14,15^. They have a gyrification degree of cerebral cortex much higher than other New World primates; and have characteristics like hominids-tool use, high encephalization, hand morphology, and dietary flexibility^15, 16, 17, 18^.

Humans have the highest encephalization quotient (7.4–7.8) among primates^16^; they are followed by capuchins with an encephalization quotient of 4.8, higher than those of chimpanzees (2.2–2.5) and gorillas (1.5–1.8)^16,17^.

Due to their complex cultural behavior and well-developed memory, the capuchin is perhaps the most intelligent primate in the Americas^13^. Foraging behavior includes locating, obtaining, processing, and eating food. The diet is based mainly on fruits (60%) but also includes other vegetables, insects, nectar, and even some species of oysters and crabs found in mangrove regions^13^.

Recent studies have shown that the bearded capuchin has extraordinary abilities^19,20^. Among them, the discovery of stone tool production demonstrates “that the production of archeologically identifiable flakes and cores, as currently defined, is no longer unique to the human lineage.”^20^. The wild bearded capuchin uses stone hammers and anvils to open hard encapsulated food, fracture wood to access insects and larvae and sticks as probes to access food, honey, and water^19^. At the mangroves, they use wooden hammers and anvils to open crabs and mollusks^14^.

Captive and wild capuchins are submitted to various cognitive tests, besides observation in a natural environment, showing a diverse capacity concerning working memory, learning and delayed recall, executive functions, and problem-solving, including a diversity of skills depending on the place of origin, and age of the monkey^21^. They have shown individual learning abilities together learning through exploratory tendencies and observation of older individuals^13–15;21–25^, including modified behavior in the consequences of cohabitation with humans in their habitat^26^.

This study aims to investigate the presence of AD-type pathology in capuchin monkeys.

## RESULTS

**Figure 1:**
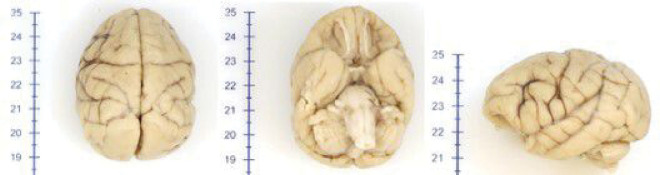
Macroscopical findings. Macroscopic evaluation of the 29 year-old capuchin monkey showing cortical gyrification.

**Figure 2:**
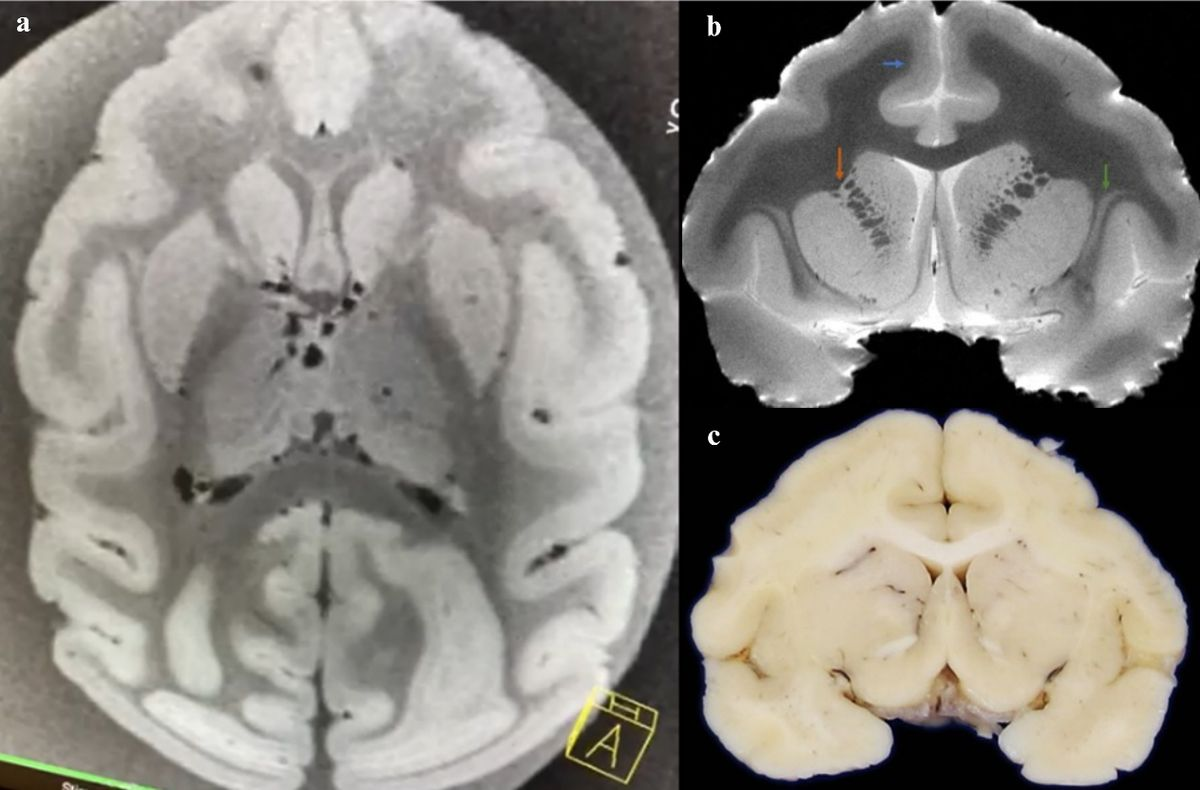
7T ex-situ brain magnetic resonance image (MRI) of 29-year-old capuchin monkey. (A) Axial brain 7T MRI showing the cortical gyrification. (B) In a T2 weighted imaging from a 7T MRI of a coronal slice, the cortical lamination of the frontal cortex (blue arrow), as well as the connections between caudate and putamen (orange arrow) were observed. Additionally, the claustrum can also be well identified (green arrow). (C) a macroscopic coronal slice at the same level of B.

Macroscopic evaluation of the brains showed considerable cortical gyrification (Fig. 1). In addition, thick cortical ribbon was observed in the MRI (Fig. 2).

### β-amyloid deposits

AD type of pathology was observed in the eldest capuchin monkey and not in the nine years old. β-amyloid (βA) deposits were observed in the hippocampus, amygdala, basal ganglia, and all cortical areas except the occipital cortex where only amyloid angiopathy was found. A moderate density of diffuse and focal immunoreactive deposits was found. Although βA deposits were observed in both hemispheres, a higher burden was noted in the left hemisphere compared to the right.

**Figure 3:**
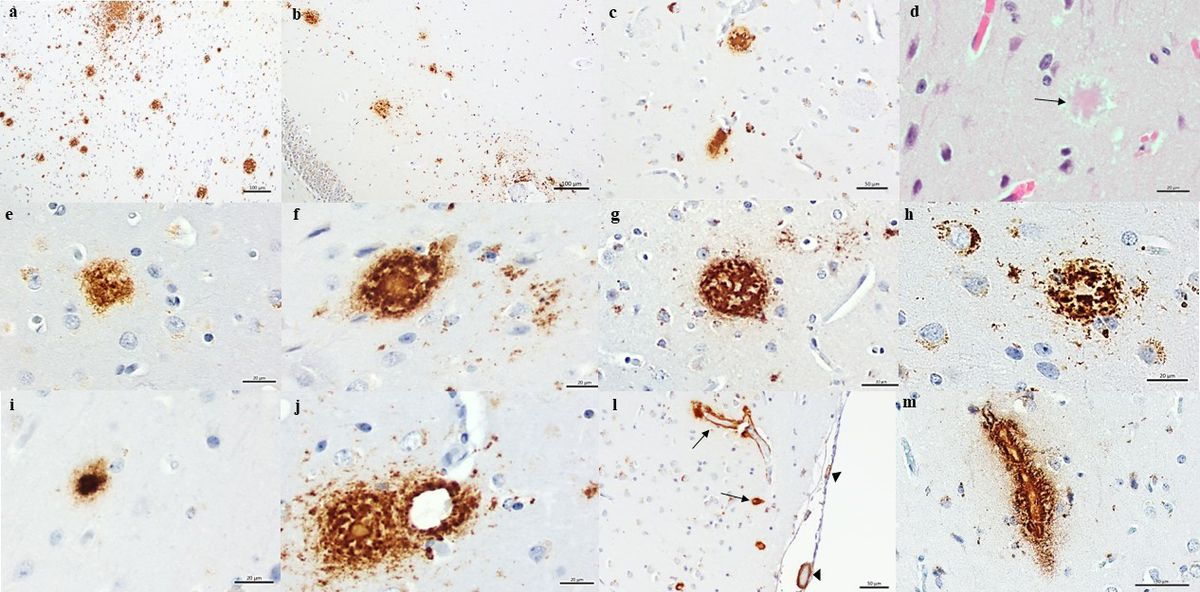
β-amyloid pathology was observed in several brain areas of 33-year-old capuchin. Immunoreactive β-amyloid deposits in frontal cortex (A), hippocampus (B), and basal ganglia (C). (D) Plaque with amyloid core (arrow) was also observed on hematoxylin-eosin staining. Focal β-amyloid deposits were observed in the form of primitive (E) and mature plaques with different morphologies (F-J). β-amyloid deposits in meningeal (arrowhead) and intracortical vessels (arrows) were observed (L) with capillaries involvement (M). 4G8 antibody (A-C, and E-L). Scale bars=100μm (A, B); 50μm (C, D, L, M); 20μm (E-J).

Widespread βA immunoreactivity was observed in the form of diffuse and focal deposits (Fig. 3). Most of them were focal with a predominance of mature than primitive plaques (Fig. 3). Notwithstanding variable plaque morphology was observed predominantly in the form of classical dense-cored plaques with core-space-corona, coarse-grained plaque, burnt-out plaque, and juxtavascular plaque (Fig. 3). Plaques with amyloid core were also observed on routine staining (Fig. 3).

In addition, in many cortical areas, βA deposits were found in the form of amyloid angiopathy in leptomeningeal and cortical vessels with involvement not only of arterioles but also capillaries (Fig. 3).

**Figure 4:**
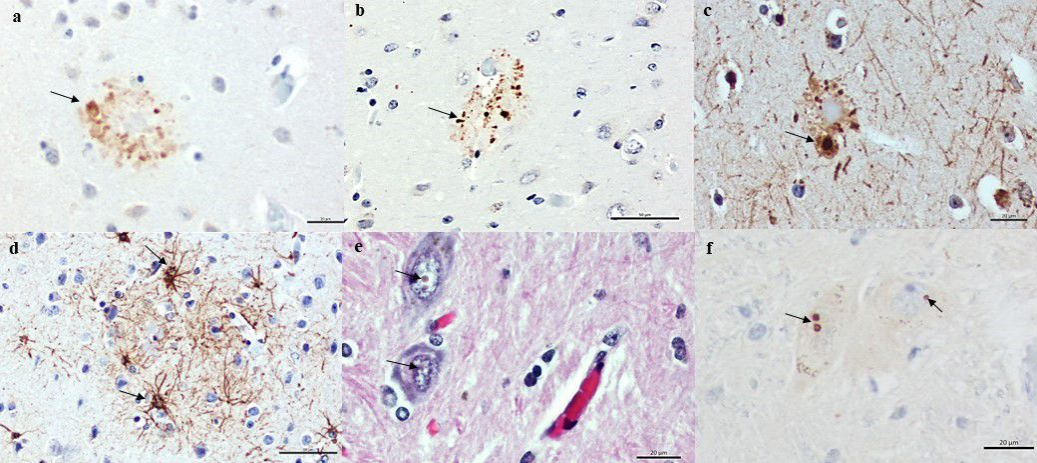
Distrophic neurites and marinesco bodies. Abnormal neurites associated with plaque (arrows) were identified with immunohistochemistry using p62 (A, B) and 2F11 (C) antibodies. Besides, hypertrophic astrocytes surrounding β-amyloid plaques were observed with GFAP antibody (arrows, D). Additionally, Marinesco bodies were found in neurons of the substantia nigra (E; arrows) and were immunoreactive for p62 antibody (F; arrows). Scale bars= 20μm (A, C, E, F); 50μm (B, D).

Additionally, plaque-associated neurites were identified using p62 and neurofilament antibodies (Fig. 4). However, hypertrophic astrocytes surrounding βA plaques were observed without microglial activation (Fig. 4).

### Hyperphosphorylated tau deposits

**Figure 5:**
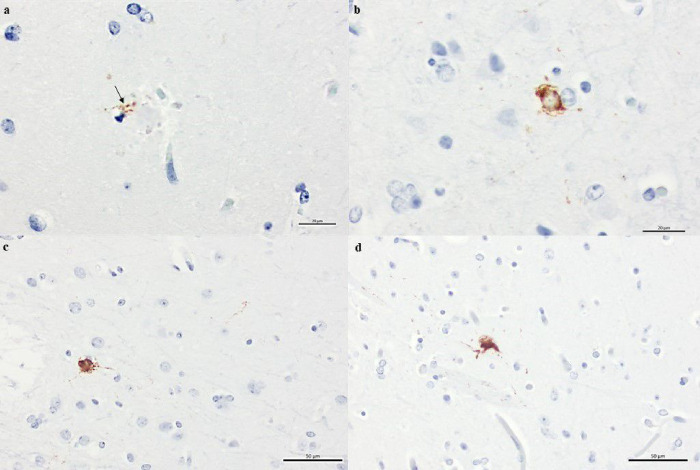
Hyperphosphorylated tau deposits observed in the form of tau immunoreactive aggregates (AT8). (A) surrounding the core of a classical neuritic plaque, (B) neurofibrillary pathology resembling a pretangle and neurofibrillary tangle (C-D). Scale bars= 20μm (A, B); 50μm (C-D).

Very few dystrophic neurites were hyperphosphorylated tau (*P*tau) immunoreactive (Fig. 5). In addition, sparse tau abnormal aggregates resembling pretangles, neurofibrillary tangles, and neuropil threads were found in the temporal and frontal cortex using AT8 antibody (Fig. 5).

### Other neuropathological alterations

Besides the presence of βA and Ptau aggregates, small intranuclear inclusions, Marinesco bodies, were observed in neurons of the substantia nigra (Fig. 4). These eosinophilic spherical nuclear inclusions were strongly immunoreactive for p62 (Fig. 4).

Immunohistochemistry using TDP-43 and phosphorylated α-synuclein antibodies failed to reveal immunoreactive aggregates of these proteins in cortical, limbic and brainstem regions.

## DISCUSSION

AD-type of pathology was found only in the brain of the eldest specimen of *Sapajus libidinosus*. Diffuse and focal βA deposits were observed in cortical and limbic areas. Noteworthy, focal deposits were more frequently than diffuse. Among them the density of mature plaques was higher than primitive plaques. This finding contrasts with previous studies describing predominance of diffuse deposits and primitive plaques in other new world monkeys such as squirrels and marmosets.

In addition, in our case neuritic plaques were frequently found not only in cortical, but also in subcortical areas such as hippocampus, amygdala and basal ganglia, contrasting with the predominance of diffuse deposits and primitive plaques described in squirrel and marmoset. Also, it is important to highlight the high density of mature plaques, even when compared to the density of CAA, and the presence of different morphologies similar to those observed in human brains in AD. Interestingly, in the eldest capuchin monkey, CAA was not the predominant form of βA pathology unlike observed in other species from the new world.

In addition, abnormal aggregates of *P*tau were observed in temporal and frontal cortex resembling neurofibrillary pathology. Although these Ptau immunoreactive pathology was sparse, they are very similar to those observed in AD brains^27^.

The lack of Ptau in the majority of dystrophic neurites surrounding the classical neuritic plaques and the sparse density of neurofibrillary pathology can represent early stages of disease^28^. However, this finding can be associated with species differences in the tau protein^7^.

Despite the low burden of Ptau pathology, the acknowledgment that these species can spontaneously develop AD-type pathology similar to observed in humans is highly relevant. Also, the presence of astrocyte hypertrophy observed indicate a neuroinflammatory response to the AD-type pathology that can contribute to neurodegeneration or play a protective role^29^. Our findings identified a nonhuman primate species that may represent a new lower primate model appropriate for AD studies.

There was no information about cognitive decline or unusual behavioral change, only decreased motor activity. It is possible that this individual was in the preclinical phase of AD. The presence of AD pathology only in the eldest Capuchin associated with a different behavior (isolation and inactivity) observed during her last months of life could indicate that AD and not advanced age is responsible for his changing.

The literature review showed that *Sapajus sp* is a primate with many qualities that recommend its inclusion among AD animal models, with some advantages to other animals. Based on genetic, behavioral, and morphological characteristics, the capuchin monkey is phylogenetically close to humans.

Compared to Old World primates, it is a relatively small animal, its maintenance in captivity is simpler and less expensive. *Sapajus libidinosus* surprised researchers with its skill in creating and using instruments^19,20^. It is a very curious and motivated animal that can be evaluated with neuropsychological tests that are similar to those used in other primates and humans^15;21–24^.

Relative to other primates such as the Saimiri^30^ and marmosets^2^ that have been proposed for animal models of AD, *Sapajus* has a much greater cerebral cortex development and greater cognitive ability^13,15,17,19,20^. Moreover, tau isoform expression pattern in marmosets may be more like that of mice than that of humans^31^.

To use monkeys as natural models of AD is a challenge due to the long time to reach old age^2^. However, the possibility of identifying cognitive decline and biomarkers of βA and Ptau pathology in these animals may be very relevant for developing evolutive biomarkers and new treatments for AD. Besides, if a nonhuman primate can develop all of the clinical and neuropathological hallmarks of AD with the introduction of a single FAD mutation, this will represent a major step in AD research^2^. It should also be considered that the Cebidae family, of which the bearded capuchin monkey is a member, is one of the less threatened primate families^32^. To conclude, *Sapajus libidinosus* should be included as an advantageous animal model for AD research.

## MATERIALS AND METHODS

In 2016, a cooperative study was launched between the Department of Neurology at the University of São Paulo (USP) and the Primate Center at the University of Brasília (UnB), whose main objectives are to provide a captive breeding colony of Brazilian neotropical primates for research ethological and biomedical. This center is located on a farm of 4.340 ha (16030” S, 46030” W) in a protected area of an ecological reserve and houses primates in cages surrounded by nearby semideciduous tropical native gallery forests under natural conditions of light, temperature, and moisture.

The housing and maintenance conditions of primates follow the Brazilian Institute of Environment and Renewable Natural Resources (IBAMA) laws and regulations. All procedures were carried out according to the Brazilian regulations for Care and Use of Animals for Scientific purposes established by the National Council for Control Animal Experimentation (CONCEA) (Lei Arouca 11.794/2008). The Ethics Committee for Animal Use from the University of Brasilia certified that all the procedures in this research are in agreement with Brazilian and international guidelines for animal experimentation (Process SEI: 23106.123230/2023–68).

Three adult capuchin monkeys of the *Sapajus libidinosus* species (02 males and 01 female) kept at the Primatology Center and who died of natural causes had their brains used for this study. The Brazilian Institute of the Environment and Renewable Natural Resources (IBAMA) apprehended the animals and sent them to the Primatology Center, arriving at that center in adulthood. Therefore, their ages are estimated and based on the morphological characteristics of the animals at the time of their arrival, plus the length of stay at the Primatology Center. They were kept in couples in cages (4m long, 2.9m wide × 2m high, per cage), which consisted of two concrete walls, separating adjacent cages and wire mesh at the front, back, and roof, forming an external/semi-internal housing system. Each cage contained a suspended wooden nest box, several wooden perches at different heights, a food tray where the animals were fed, and a thick layer of natural dry leaves on the floor. The animals had olfactory and acoustic contact between colony members but not visual contact. Their food was based on fresh fruits and vegetables and was provided daily from 7:30 a.m. until 5:00 p.m. Primate feed and fresh water were provided *ad libitum*. The animals received permanent veterinary monitoring and underwent clinical evaluations and monthly weightings.

So far, complete neuropathological evaluation was available for two cases (9 and 33-year-old males). Meanwhile, a structural 7 Tesla magnetic resonance image (7T MRI) was acquired in one case (a 29-year-old female) after and before brain slicing (see Table 1). S2, the oldest male animal, may have a possible cognitive decline, as evidenced by his inability to reach the learning criterion in a visual discrimination test carried out in 2014 despite successive training sessions. However, it is worth noting that S2 performed comparably to other adult animals in a working memory protocol (delayed matching and non-matching-to-sample tasks) twelve years earlier^21^. Additionally, this animal showed a decrease in motor activity, usually seen in primates with aging, which may also suggest some age-related decline in his overall health and functioning.

Deaths were due to natural causes, and an experienced veterinarian performed the brain extraction. Brain tissue was fixed in 4% buffered paraformaldehyde within 12 hours of death for three weeks. Macroscopic evaluation, photographs, and postmortem 7T MRI were performed on one individual. Consecutive coronal sections from the fixed brain were embedded in paraffin, and 5μm sections from paraffin blocks were used for staining and immunohistochemistry evaluation. All brain sections were stained with hematoxylin and eosin.

Immunohistochemistry with antibodies against β-amyloid (4G8, 1:10.000; Biolegend), tau phosphorylated at Ser^199–202^-Thr^205^ (AT8, 1:400; Thermo Fisher), p62 LCK ligand (p62, 1:500; BD Bioscience), phosphorylated transactivation response DNA-binding protein of 43 kDa (TDP-43, 1:500; Biolegend), α-synuclein phosphorylated at Ser^129^(81A, 1:500; Biolegend) 68 kDa neurofilament (2F11; Sigma), glial fibrillary acidic protein (GFAP; Dako) and microglia (CD68; Dako) were performed in selected sections of both hemispheres to analyze the following areas: frontal cortex, temporal cortex, parietal cortex, occipital cortex, anterior cingulate cortex, hippocampus, amygdala, basal ganglia, thalamus, mesencephalon, pons, medulla oblongata, and cerebellum.

## Figures and Tables

**Table 1:** Characteristics of the sample

Identification	Sex	Age at death (years)*	7T MRI **	AD-type of pathology
Sl.1	Male	9 (adult)	No	No
Sl.2	Male	33 (older capuchin)	No	Yes
Sl.3	Female	29 (older capuchin)	Yes	Not investigated

* Estimated age of death;

** postmortem

## Data Availability

All data are available in the main text.
